# Preparing Europe for future health threats and crises – the European Health Emergency and Preparedness Authority; improving EU preparedness and response in the area of medical countermeasures

**DOI:** 10.2807/1560-7917.ES.2022.27.47.2200893

**Published:** 2022-11-24

**Authors:** Pierre Delsaux

**Affiliations:** 1 European Commission, Health Emergency Preparedness and Response Authority (HERA), Brussels, Belgium

**Keywords:** European Health Union, Health Emergency Preparedness and Response Authority, HERA, preparedness, health crises, medical countermeasures

On 24 October 2022, the adoption of the Regulation on serious cross-border threats to health, the Regulation on the extended mandate of the European Centre for Disease Prevention and Control (ECDC), and the Council Regulation on the emergency framework regarding medical countermeasures (hereinafter, the Emergency Framework Regulation), which provides extra powers to the European Health Emergency Preparedness and Response Authority (HERA), concluded the creation of the European Health Union [1]. Other building blocks of this new EU health security framework are the Regulation on the extended mandate of the European Medicines Agency (EMA) adopted in March 2022 [2] and the creation of an entirely new permanent structure for health emergency preparedness and response. Established within the European Commission (EC) as a shared resource for both the European Union (EU) and its member countries, HERA was set up in September 2021 to further strengthen Europe’s ability to prevent, detect, and rapidly respond to cross-border health emergencies by ensuring the development, manufacturing, procurement and equitable distribution of key medical countermeasures. HERA’s governance ensures an extensive coordination and cooperation with EU countries via the HERA Board and the HERA Advisory Forum. In addition, structured exchanges with representatives of the civil society and industry are organised through subgroups of the HERA Advisory Forum.

With a budget of EUR 6 billion for the 2022–2027 period, HERA‘s mandate covers all serious cross-border health threats and related medical countermeasures, including crisis-related vaccines, medicines, personal protective equipment (PPE), diagnostics, active pharmaceutical ingredients (API) and critical raw materials. To further target its preparedness activities, in 2022, HERA has conducted a thorough threat prioritisation exercise, in consultation notably with ECDC and EMA, which resulted in the identification of the following three threat categories, namely: (i) pathogens with high pandemic potential; (ii) chemical, biological, radiological, and nuclear (CBRN) threats originating from accidental or deliberate release; and (iii) antimicrobial resistance (AMR) [3]. The inclusion of AMR in the top three priority threat categories shows that HERA does not aim solely at preventing or addressing acute public health events, but also more slowly evolving, silent pandemics with high associated public health burden.

In full respect of the subsidiarity principle, HERA operates in two modes: the preparedness mode to anticipate and respond to threats before they turn into crises, and the crisis mode empowering HERA to coordinate and take action against health emergencies. This differentiation allows for a high level of flexibility as well as extended coordination and cooperation with EU countries, other EC services and EU agencies, as well as external actors to contribute to the identification of EU priorities, mutualise efforts and leverage resources.

As long as the emergency framework as defined by the Emergency Framework Regulation is not activated [4], HERA operates in preparedness mode in synergy with EU countries, ECDC and EMA, and international partners. Activities focus on intelligence gathering and threat assessment; promoting advanced research and development of medical countermeasures; and ensuring sustainable access to medical countermeasures through the coordination of purchase, manufacturing, and stockpiling activities.

Intelligence gathering and threat assessment encompasses both short- and long-term activities, including the continuous monitoring and assessment of signals to rapidly identify the health events requiring medical countermeasure response. The function also covers horizon scanning to guide policy, research, and innovation planning as regards medical countermeasures. In addition, HERA is developing a comprehensive mapping of medical countermeasures that are critically important to address the three identified priority threats. This mapping will help HERA rapidly draw the list of crisis-relevant medical countermeasures to be established in case of the activation of the Emergency Framework Regulation.

To support HERA’s intelligence gathering and threat assessment function, the HERA IT platform is currently being developed. It will be a highly accurate digital system which will make use of a wide range of data to guide decision-making on the medical countermeasures response, including by forecasting, developing and testing specific scenarios. Data processed by the HERA IT platform will include the outputs of the networks for epidemiological surveillance and the epidemic intelligence systems operated by ECDC, as well as forecasts and information gathered by EMA on existing medicines, including shortages, and those in development.

Via Horizon Europe and EU4Health funding, HERA is further investing in pandemic preparedness research and development, for instance by setting up sustainable networks, platforms and infrastructures that can be adapted quickly to emerging or previously unknown pathogens. As one example, the pandemic clinical trials platforms that were set up by the EC during the COVID-19 pandemic on vaccines and therapeutics (VACCELERATE [5] and EU-RESPONSE [6], respectively) will be further developed. Coordination mechanisms between the different clinical trials within the platforms are being established, and close cooperation with the EMA-Emergency Task Force (ETF) [7] is foreseen for scientific, regulatory and technical guidance regarding protocol design as well as coordination for larger, multinational clinical trials. Further integration of clinical research in threat preparedness is envisaged by the targeted development of and access to specific medicinal products with the potential to improve preparedness and response to serious cross-border threats to health, such as broad-spectrum antivirals and a new generation of COVID-19 vaccines.

Rapid and equitable access to medical countermeasures is essential in responding to health emergencies. At any time, HERA can mobilise EU funding to organise and coordinate development, manufacturing and procurements (including stockpiling) of relevant medical countermeasures. Through the COVID-19 Vaccines Strategy [8], the EC was able to speed up the development and manufacturing of safe and effective COVID-19 vaccines, and to secure up to 4.2 billion doses of COVID-19 vaccines. In addition to the vaccine contracts, a number of framework contracts for COVID-19 therapeutics were put in place to facilitate access to those for the EU countries. HERA continues to manage all these contracts and also carries out horizon-scanning for new vaccines or therapeutics which may be of interest to countries.

A new mechanism to improve Europe’s overall preparedness for response to health crises established by HERA is the EU FAB. This network of facilities ready to be operational at all times for vaccine production in the EU and  European Economic Area (EEA) can be activated in case of a health emergency to scale up production of different types of vaccines.

As regards stockpiles of medical countermeasures, HERA is defining a coordinated strategy to enhance their effectiveness and sustainability. In parallel, HERA is building stockpiles under rescEU to address high impact and low probability threats [7], such as CBRN events, with relevant medical countermeasures. A first call for proposal was launched in March 2022 and was further expanded in light of the current geopolitical situation.

HERA has a strong international dimension, through which it contributes to reinforcing global health security while also ensuring availability and access to critical medical countermeasures for all. This includes engaging with strategic global partners, such as the United States Biomedical Advanced Research and Development Authority, the World Health Organization Hub for Pandemic and Epidemic Intelligence, Coalition for Epidemic Preparedness Innovations, and Africa Centres for Disease Control and Prevention among others, to reinforce global surveillance and intelligence gathering, address international supply chain bottlenecks and expand global production capacity.

The Emergency Framework Regulation provides the basis for HERA to shift into crisis mode. The declaration of a public health emergency at EU level could trigger the activation of the emergency framework and allow for increased coordination and enable HERA to take necessary measures for sufficient and timely availability and supply of crisis-relevant medical countermeasures. To be effective and operational in times of public health emergencies, a new configuration will be rapidly convened within HERA. The so-called Health Crisis Board, co-chaired by the EC and the country holding the rotating presidency of the Council of the EU, will guide decision-making on medical countermeasures. EMA and ECDC will be represented by their respective Directors and will play a crucial role. EMA will regularly report information on the monitoring of medicinal products and medical devices, including their demand and supply, and ECDC will provide information on epidemiological surveillance outcomes and countries’ health system capacity.

Based on the information collected, HERA will provide a comprehensive overview of the needed crisis-relevant medical countermeasures and the EU’s capacity to meet those needs. While HERA and EMA will both have the mandate to monitor supply and demand of certain medical countermeasures in the event of public health emergencies, their focus will differ based on the respective expertise. In particular, EMA’s monitoring of supply and demand will focus on selected authorised critical medicines, including the gathering of information on their API, and certain types of medical devices. HERA will complement this monitoring by covering a broad range of crisis-relevant medical countermeasures, including both authorised and unauthorised medicines and medical devices, but also raw materials, diagnostics and PPE. Using the intelligence gathered, HERA would then advise on the activation and the implementation of the EU FAB facilities, emergency research and innovation plans and access to emergency funding.

HERA, ECDC and EMA work closely to leverage resources and expertise across their respective mandates and to contribute to a proportionate and efficient crisis response within Europe and globally. While HERA is the major player in the identification, development, procurement, stockpiling and deployment of medical countermeasures for health emergencies, this area also requires close collaboration between HERA, ECDC and EMA. HERA builds on and supplements the activities carried out by the ECDC and EMA on monitoring, prevention, and control of serious cross-border health threats and on development and authorisation of medical countermeasures.

For example, HERA uses the outputs of ECDC activities – notably from the networks of epidemiological surveillance, the ECDC epidemic intelligence system, and the ECDC risk assessments as regards communicable diseases and threats of unknown origin – and combines them with outputs of other surveillance systems and its own data collection in order to rapidly identify threats requiring a medical countermeasures response.

Further, independent of the special mode of cooperation in public health emergencies as described above, in preparedness time HERA closely cooperates with EMA on availability of medicines identified as potential countermeasures as well as to identify products not authorised in the EU and products in development that could be potentially of interest in case of public health emergencies. HERA complements the information received from EMA with its own intelligence gathering. Conversely, the outputs of HERA’s intelligence gathering and threat assessment relevant to medical countermeasures are shared with ECDC and EMA to feed ECDC’s risks assessments and guide EMA scientific advice and regulatory support for the relevant medical countermeasures.

The rapid and coordinated response to the ongoing monkeypox outbreak in Europe has shown that HERA, ECDC, and EMA have set up mechanisms to avoid duplications and ensure efficient cooperation across their respective mandates (Box).

Box **The case of monkeypox – an example of the close collaboration of HERA, ECDC and EMA**

After the ECDC epidemic intelligence picked up signals of a monkeypox outbreak in the EU in May 2022 [9], HERA, advised by EMA, identified the available authorised and unauthorised treatments and vaccines for monkeypox. Only one authorised therapeutic and only one vaccine was available to address this emerging threat in the EU. Thus, in accordance with its mandate and after consultation of the HERA Board, HERA then collected, on a voluntary basis, data from national competent authorities and the industry on supply and demand for the therapeutic and the vaccine. The intelligence gathered allowed HERA to rapidly purchase over three hundred thousand vaccine doses and donate them to EU/EEA countries, as well as to organise joint procurements for purchasing antivirals. Looking ahead to the medium and long-term needs, HERA signed a Joint Procurement Framework contract for the supply of up to 2 million doses of the monkeypox vaccine during 2023 and 2024 [10].

EMA provided scientific opinion and recommendations regarding the safety and quality of the purchased monkeypox vaccines and antivirals as well as a recommendation on the dose-sparing administration of monkeypox vaccines [11]. ECDC organised surveys on vaccination strategies and vaccine acceptance which provided evidence to assess and update the needs for vaccines in EU countries [12] and adjust HERA’s purchase and procurement activities accordingly.

Once the outbreak was declared a public health emergency of international concern by the World Health Organisation [13], both drugs were included in the list of critical medicines established by EMA, which then organised regular and mandatory collection of supply and demand data. EMA could swiftly implement this data collection, building on the work carried out by HERA in preparedness time to collect similar data, but on a voluntary basis, for the purpose of its procurement activities.

To ensure a common approach, HERA and EMA jointly discussed with countries in the HERA Advisory Forum a European strategy for additional clinical data collection on the safety and efficacy of monkeypox treatment and vaccines. Scientific advice on clinical trial protocols was provided by EMA Emergency Task Force. The EC initiated the mobilisation of Horizon Europe emergency funding to support two complementary, multinational therapeutic clinical trials, which were submitted in the Clinical Trials Information System under the Clinical Trials Regulation (EU/536/2014) and that use the EU-RESPONSE pandemic trial platform.

One year after the creation of HERA and with the completion of the European Health Union, the EU has better tools, capacities and structures to respond to health crises. The EU pandemic preparedness and response system needed a fundamental transformation. The EC and the co-legislators have– judiciously and in full respect of the EU countries’ competences – addressed this need by creating a legislative framework and reinforced permanent structures to face the challenges ahead. HERA, ECDC and EMA are at the heart of this new and still evolving set-up. Their concerted and determined efforts, in close partnership with countries, will make the EU better prepared for and able to respond quickly to future cross-border health threats.

## Note

Pierre Delsaux is the Director General of the Health Emergency Preparedness and Response Authority (HERA).
